# On the Resuscitation of Infants Born Still

**Published:** 1860-05

**Authors:** William C. Rogers

**Affiliations:** Green Island, Albany County, N. Y.


*On the Resuscitation of Infants Born Still.* By William C. Rogers, M.D , Green Island, Albany County, N. Y. Second Article.

In the writings of standard obstetricians, and in the periodical literature of the profession, I have found, up to the publication of my first article, no analysis of the various conditions of the still neonatus, and I accordingly sought to supply this want. I accumulated my material, published my first article, and was reading and reflecting for my second, when Dr. T. Gaillard Thomas, of New York, kindly sent me a copy of his valuable "Lecture on Suspended Fœtal Animation," in which he throws the clear light of science upon the still neonatus, and thereby increases the resources of our art in the management of these important and trying cases. As he has thus anticipated me in my labor, I shall make free use of the materials he has placed in my hands, hereby acknowledging my indebtedness to him, and shall add thereto that which my reading, reflection, and experience have led me to consider pertinent and valuable.

1. *Of the Conditions of the Still-Born Child.*—The neonatus may suffer from—*a*, Asphyxia; *b*, Syncope; *c*, Cerebral Congestion; and from, *d*, Apoplexy. (Thomas.)

### *a.* Of Asphyxia.

Asphyxia is a suspension of the function of respiration, independent of the motions of the heart, or of the circulation of the blood. In *asphyxia neonatorum*, placental respiration is either imperfectly performed or has entirely ceased, while pulmonary respiration has not been established. As a consequence, the blood becomes rapidly surcharged with carbonic acid and the detritus of the fœtal system, and is propelled to the lungs. Here the normal reactions between the blood and its surroundings cannot take place, because of the absence of atmospheric air from within the pulmonary vesicles, and the abnormal condition of that fluid within the pulmonary capillaries; and hence arises accumulation of blood within the venous, and diminution of blood within the arterial system, with congestion of the lungs, and of the right side of the heart. The carbonized blood, circulating in the brain and nervous centres, feebly stimulates or utterly paralyzes them; no nerve-force is evolved, respiration is not established, and we may have for a season active motion of the heart, and circulation of venous blood without coincident respiration.

Such is, in brief, the physiology and pathology of asphyxia. Its causes may be dependent upon, 1st, the condition of the mother; 2d, upon the condition of the uterus and placenta; 3d, upon the condition of the cord; and 4th, upon the condition of the child.

1st. *Causes Dependent on the Condition of the Mother.* The principal of these is *maternal toxæmia.*

The maternal blood, passing from the uterine into the placental sinuses, washes the vast mass of fœtal tufts of which the placenta is formed, and by the processes of endosmose and exosmose, of exhalation and absorption, absorbs carbonic acid and the detritus of the fœtal system from, and imparts oxygen to, the blood of the fœtus. The placental circulation is thus virtually respiration, analogous in all essential respects to the bronchial respiration of the fish; since the maternal blood, containing oxygen, in washing the fœtal tufts of the placenta, performs the same functions for the child that the water containing atmospheric air, and washing the bloood-vessels of the bronchiæ, performs for the fish. And it is evident that the analogy may be traced still further. If the water in which the fish is respiring is not renewed, its oxygen is soon exhausted, and the fish dies asphyxiated; and in like manner, if the maternal blood be not depurated, or if it be not renewed from any cause, asphyxia and impending death is the result. When the mother's blood is not depurated we have maternal toxæmia or uræmia—a condition characterized by the presence in the blood of urea, or the carbonate of ammonia,* in abnormal quantities. This uræmic condition of the mother's blood gives rise to puerperal convulsions, which are even more fatal to the fœtus than to the mother. Of 185 cases of puerperal convulsions cited by Churchill and his American editor, 47, or about *one-fourth*, were fatal to the mother; while Braun estimates the infant mortality in this disease at 45 per cent. Ramsbotham records 18 cases of puerperal convulsions occurring before delivery in 48,682 deliveries, the infant mortality of which was 12 in 18. Dewees records 8 cases in which 4 children were born living, 2 dead, and in the remaining 2 the mothers died undelivered. Collins records 30 cases in 16,654 deliveries, in 14 of which the children were born dead, 2 were putrid at birth, and 14 were born living. What proportion of these cases depended upon uræmia we have no means of knowing, nor have I anywhere seen an estimate of the probable proportion of cases of puerperal convulsions in the hundred depending upon this condition, but it is undoubtedly very great. The causes of the great fœtal mortality in this fearful complication of labor are, 1st, circulation of carbonized blood through the placental sinuses, gener-

* A résumé of all that is known of uræmia is much needed. The whole subject is so little understood, the results of the experiments of Frerichs, Richardson, Hammond, and others, are so contradictory, and the nature of the disease so obscure, that we are scarcely warranted in affirming anything of it positively, beyond its mere phenomena. These are not sufficiently developed to admit of comprehensive generalization; and until such generalization is possible and satisfactory, we are not warranted in arguing from our present theories as though they were truths, comprehending and satisfying all the relations of all the phenomena.

ated by the arrested or imperfect respiration of the mother; 2d, absorption of urea and the carbonate of ammonia from the maternal into the fœtal circulation; (?) and 3d, partial or complete closure of the uterine sinuses by the spasmodic action of the abdominal muscles.

Statistics would, without doubt, show a greatly diminished maternal and fœtal mortality in uræmic convulsions since the use of chloroform in this disease, but whether or no this diminished mortality could be attributed to the effect of the chloroform in preventing the decomposition of urea into the carbonate of ammonia by producing a temporary diabetes, as Prof. Simpson supposes, must be regarded still as a very open question. Braun makes the following assertion: "If, after numerous uræmic convulsions, the child is born alive, a large quantity of urea is found in the blood taken from the umbilical cord; but if it is born dead, we can immediately after the birth demonstrate the presence of carbonate of ammonia in the fœtal blood."

Tyler Smith says, "It is found that children born alive after (puerperal) convulsions are affected with uræmia or albuminuria, and that this condition lasts in some cases for a considerable time after birth. Sometimes children born alive under such circumstances have themselves died subsequently of uræmic convulsions." "A large proportion of the children in such cases are born dead." (*Obstet.*, Gardner's Edit., p. 623.)

An interesting case, illustrating the effects of a toxic condition of the mother's blood upon the fœtus, is recorded by Dr. W. H. Thayer in the *Trans. N. H. Med. Soc.* for 18[illegible]7, p. 94. Dr. Thayer was called in consultation in the case of a woman who had been in labor 38 hours; he administered ether, and delivered by the forceps. The child was born still, and resisted all efforts at resuscitation for ten minutes; the umbilical cord was then cut, and allowed to bleed a few jets before the ligature was applied; efforts at resuscitation were renewed, and in ten minutes more were successful, when "*the breech of the infant had a decided odor of ether.*" The mother returned to consciousness about the time the child was resuscitated, thirty minutes after the ether was discontinued. The mother was fully etherized at the time the umbilical cord was cut; the placental circulation was not suspended; the establishment of respiration followed shortly after the division of the cord, having been delayed "by embarassment of the circulation due either to hyperæmia or the presence of ether in the blood."

2. *Causes Dependent upon the Condition of the Uterus and Placenta.*—These causes are, firm and abnormal contraction of the uterus, whereby the uterine and placental sinuses are closed; partial and hour-glass contractions of the uterus, and partial or complete separation of the placenta.

Of the first of these, Dr. Thomas remarks: "After a labor has lasted many hours in the second stage, or after the adminstration of ergot at any period of the stage, the uterus not only exerts its contractile powers by alternate and intermitting contractions, but closing firmly, as it were spasmodically, upon the child, it enters into a constant and unintermitting contraction, the power of which they can testify to whose arms have been nearly paralyzed by the performance of the operation of version. Now, this firm and unnatural contraction of the organ presses the spongy mass, the placenta, firmly against the child's body, closes its pouches, in which the mother's blood flows over the fœtal tufts, and obliterates the uterine sinuses through which that blood passes to the placenta. It is, in fact, only an exaggeration of that state which we produce by rupturing the bag of waters to check hæmorrhage in the first stage of labor; and exactly the same principle is developed that we employ when we bind a billiard-ball firmly in the palm of the hand to check hæmorrhage from the palmar arch."

Dewees devotes five pages to the consideration of "Partial Contractions of the Uterus," in which he shows that the os may contract firmly about the neck of the fœtus during labor, thereby not only delaying the progress of labor, but imperiling the child by the induction of asphyxia. I have met with no cases on record where this latter result has been ascribed to this cause, but a moment's reflection will show us that it might very easily occur; and in cases where the labor seemed delayed from other than the ordinary causes, such as rigidity of the soft parts, insufficient pains, &c., a more careful examination might discover this condition of the organ. The same remarks will apply with equal force to hour-glass contractions of the uterus, whereby the body of the fœtus is so pressed by the contracting segment of the organs, as to arrest circulation in the cord, to induce cerebral congestion, apoplexy, or fatal asphyxia. Records of cases of this kind I have found in my reading, but, having lost the references to them, I am unable to re-record them in this place. The following case of very recent occurrence is in point:

A professional friend of mine was recently called in consultation in a case of contracted pelvis, the child's head, though of the usual size, but of unusual firmness, being too large to admit of natural delivery. He rectified the position, and waited in vain for the natural efforts to complete the delivery. He then applied the forceps, but with no better success. He then performed craniotomy, and with the crochet in the foramen magnum of the occiput endeavored in vain to deliver the mutilated fœtus. The hand introduced into the uterus revealed an hour-glass contraction of that organ, firmly embracing the child's abdomen, and effectually resisting all his exertions with the forceps and crochet. He gradually dilated the stricture, and effected the delivery.

The manner in which partial or complete separation of the placenta produces asphyxia neonati is evident, and needs but little elucidation at this time. In this condition of the placenta, whether it be entirely within the uterus, or partially or wholly over the os, the oxygenated maternal blood flows from the placenta without decarbonizing the fœtal blood, and asphyxia is the certain result.

3. *Causes Dependent upon the Condition of the Umbilical Cord.*—These causes are compression and rupture of the umbilical cord, and twisting of the cord about the child's neck. Compression may be produced by prolapse of the cord, or by interposition of the cord between the child's body and the contracting uterus. In prolapse of the cord the child's life is jeopardized by the compression of the cord between the presenting part of the child and the bony or soft parts of the mother; fortunately this complication of labor, so fraught with danger to the child, is not of frequent occurrence. I have gathered from the standard and periodical literature of the profession, and from my own personal friends and correspondents, a record of 88,342 presentations, of which number 285 were presentations of the cord: Ratio, 1 in 310. Of the fatality accompanying these 285 presentations of the funis, I had no means of ascertaining, as it was in but few instances recorded; but the following facts and figures, which I have accumulated from the sources indicated above, will throw light upon the question:

| | | |
|---|---|---|
| Whole number of cases collected, . . . . . | | 234 |
| Unassisted labor, children born living, . . . | 54 | |
| " " " " dead, . . . . | 83 | |
| Version performed, and " " living, . . . | 14 | |
| " " " " " dead, . . . | 36 | |
| Forceps cases, " " living, . . . | 13 | |
| " " " " dead, . . . | 6 | |
| Twin " " " living, . . . | 18 | |
| " " " " dead, . . . | 10 | |
| Children born living, 99. Dead, 135. | Total, | 234 |

Ratio of the dead to the living about $2\frac{1}{3}$ to 1.

It is my intention to present these statistics in full to the profession as soon as present engagements will permit.

Of rupture of the umbilical cord as a cause of asphyxia neonatorum I have found many recorded cases, but, unfortunately, have mislaid my notes and references. The majority of these cases occurred in the earlier months of pregnancy; and those which occurred during the later months, when the child is considered viable, gave evidences of fatty or other degeneration of the cord and placenta, and were accompanied by the birth of illy-developed, and doubtless non-viable, children. The further consideration of this cause, then, promises but few useful results in this connection.

The twisting of the cord about the child's neck is a frequent cause of asphyxia. Nos. 16 and 22 of Article first are cases in point, and the experience of every practitioner will furnish many such.

4. *Causes Dependent upon the Condition of the Child.*—These may be a natural feebleness of constitution, incapacitating the child for the spontaneous muscular exertion necessary to establish respiration, or a similar incapacity produced by long-continued pressure upon the brain, (Collins,) and an accumulation of mucus or liquor amnii in the trachea, pharynx and mouth, so great and so tenacious as to resist the child's feeble efforts at respiration or dislodgment. The first cause is frequently active in premature children of the seventh and eighth month; the last to a greater or less extent in almost every child at birth.

*Symptoms.*—" The fauces of the asphyxiated child is eloquent in telling of the lesions; the face is dusky, the lips purple and pouting, the eyes glassy, and the limbs unyielding, and not flaccid, as in syncope. The heart may be felt beating feebly, or not at all; or its strokes may be intermittent, while there is not the slightest effort at respiration."—(*Thomas.*)

*Prognosis.*—The prognosis will depend, in a very great measure, upon the condition of the heart. If that organ continues to pulsate ever so feebly, our efforts to resuscitate *may* be successful, and should be continued as long as the heart is in action. This is illustrated by the cases adduced in the first article, twenty-four in number, in which the average period intervening between birth and the establishment of respiration was 35 minutes, 30 seconds. The seven exceptional cases adduced therein, in which respiration was not accomplished in periods ranging from 20 minutes to 7 hours, increase the average to 50 minutes of suspended fœtal animation. Mr. Tompkins, a former student of Dr. Blundell, reported a case to that gentleman, of a child recovered under the use of resuscitants, continued for a period of one hour and five minutes before obvious signs of life appeared.—(Blundell, p. 117.) The prognosis, then, may be regarded as favorable as long as the heart is in action; unfavorable the instant that organ ceases to beat.

*Treatment.*—The indication in the treatment of asphyxia is to excite the function of respiration, by which means the normal relations existing between the pulmonary capillaries and their contained blood may be restored, or rather established. The blood yields up its carbonic acid to, and absorbs oxygen from, the air; pulmonary circulation begins, and pulmonary congestion diminishes, *pari passu;* the general circulation is re-established, nervous force is elicited from the centres, and the infant begins its independent life. The great desideratum, then, is respiration. How shall it be established ?

The experiments of many observers seem to prove that the liquor amnii penetrates during intra-uterine life as far as the bronchiæ, (Velpeau.) Hence the necessity of cleansing the trachea, pharynx, and mouth, by placing the child's mouth downward, with the body and hips higher than the head, and by gentle shakes disengaging the contained fluid, or by the finger, previously protected by a soft rag, introduced well into the pharynx. This is to be premised when necessary, no matter what the condition of the child, or what means are to be used for its resuscitation. The minor remedies for asphyxia are now to be tried, such as frictions, blows upon the nates, back and thorax, irritation of the nares and throat, exposure to draughts of cold air, and like measures, calculated to awaken nervous energy and excite reflex actions. These, in the majority of instances, are sufficient; but should they fail, recourse must be had to artificial respiration, which may be executed by the mouth-to-mouth process, by the tracheal pipe, by manipulations of the chest, or by the Ready Method of Marshall Hall.

In the mouth-to-mouth process, the accoucheur's mouth is to be placed to the child's mouth, the nares are to be closed, and gentle pressure made upon the larynx, so as to close the œsophagus by pressing it against the cervical vertebræ, that no air may enter the stomach; and then the child's lungs are to be gently, but forcibly, inflated, and allowed to empty themselves by their own elasticity, or by gentle pressure upon the thorax and abdomen.

The tracheal pipe is to be introduced along the index-finger of the left hand, previously inserted into the rima, and used as a director. Examination in front of the neck will show whether the tube be in the trachea or not. If in, the infant's lungs are to be slowly, gently, and yet forcibly inflated, and emptied, as in the process above stated.

There should be from 20 to 30 respirations a minute, corresponding in number to the respirations of the quick neonatus. Dewees used this means of exciting respiration for nearly forty years, with almost uniform success. Velpeau recommends the use of a quill-barrel, a female catheter, any kind of canula, or a simple gum-elastic catheter. In the first article will be found four successful cases of its use reported by J. Toogood, Esq. Next to the Ready Method, this is unquestionably the most valuable means at our disposal for the restoration of suspended fœtal animation.

Another process is the alternate elevation and depression of the thorax by the fingers inserted well under the edge of the ribs. Dr. Pitcher, of Hudson, informs me that he has used this process to the exclusion of all others for many years, and with such a measure of success that he is not inclined to abandon it for any other. It commends itself for its simplicity and the ease with which it may be executed.

It now remains to consider the Ready Method of Marshall Hall, which, since its first successful adoption, in February, 1857, has practically superseded all others. The method itself I need not describe. It is only necessary to clear the trachea and pharynx, draw the tongue well forward, and rotate the child, *s. a.* from 20 to 30 times in the minute. Of all the methods which I have used, this is by far the most successful. A half dozen rotations will suffice in ordinary cases to remove profound asphyxia. Eleven of the 24 cases recorded in article first were restored by this method, after an average duration of suspended animation of 27 minutes. In the course of my reading, I have found but one practitioner who prefers the mouth-to-mouth process to the Ready Method, and that is Dr. A. T. Keyt, of Cincinnati, who, in the *Lancet and Observer*, for January, 1860, presents the following in substance: The still neonatus has never respired; its air-vesicles have never been opened; its chest has never been expanded; its chest and lungs are, then, devoid of elasticity; and its condition is not justly analogous to that of the asphyxiated adult. The capacity of its chest could be but little, if any, diminished or increased by the rotation process. He therefore prefers the mouth-to-mouth process, and reports cases of comparative trial. In one he says: " At least an hour elapsed before the child gave a gasp, and two hours before it could be left to do its own breathing. My dependence was upon the *mouth-to-mouth* process; by it I found no difficulty in controlling the circulation. It seemed as though the heart's action might have been thus maintained indefinitely. The 'Marshall Hall Method' was tried, but the results were negative; under it pallor and lividity would return to the surface, and the circulation grow gradually more and more feeble, until the heart's action would plainly have soon ceased, had it not been timely aroused by a more *ready* and *efficient* method. Several times did I alternate the new method with the old, and just as often did I witness the same striking contrast of phenomena." (Quoted from American Medical Monthly, March, 1860, p. 200.) This is a very interesting experience, and commends itself to the profession. Its lesson is, rely not too exclusively upon any *one* method of resuscitation, but be completely armed by being practically familiar with all. But, while Dr. Keyt's theoretical argument against the Ready Method is well grounded so far as his cases and experience go, it is not equally well grounded upon the cases and experience of others. This is evident from the eleven cases adduced in my first article, and from experiments first made by Mr. Hall himself, and afterwards repeated by Dr. Thomas before his class and detailed in his lecture, so frequently referred to. The mouth and one nostril of a still-born fœtus were closed securely by adhesive plaster, and in the other nostril a caoutchouc tube was inserted, having a bent glass tube containing water attached to its outer end. Pronation and rotation were then performed, according to M. Hall's directions, when bubbles of air would rush through the water to and from the lungs.

Here is a demonstration of the fact, that in *some* instances at least the still neonatus may be made to breathe, though *theoretically* its chest and lungs are devoid of the elasticity so requisite for the performance of this method. In my opinion, Dr. Keyt errs in condemning the Ready Method too emphatically in such cases, while Dr. Thomas errs in asserting too confidently that this is the *only* method by which artificial respiration can be effected. While I regard this method as by far the best and most philosophical yet devised, (since it introduces pure air into the child's lungs, and not the carbonized air of the accoucheur's lungs,) I prize the other methods adduced above as valuable beyond all price, and not to be discarded on any considerations. In this, as in other matters, professional and extra-professional, *in medio tutissimus ibis*.

I have said nothing as yet of the warm and cold baths so generally recommended and used in the resuscitation of infants born still. A reference to the nature of asphyxia shows that it is of the utmost importance to know which to use, the warm or the cold bath, and which to discard. In this state we have congestion of the lungs, repletion of the venous, with partial emptiness of the arterial system, and a languid circulation of carbonized blood. By establishing artificial respiration we induce the normal chemical changes in the bloood, relieve the congestion of the lungs, replace the venous with arterial blood in the systemic circulation, and thus relieve the general venous congestion. But in conducting this process we must be careful to preserve the true relations existing between respiration and circulation. Should the circulation not increase *pari passu* with the respiration, we have a constant loss of temperature from within, and no alleviation of the symptoms. Our remedy in this state of affairs is the warm bath, whereby the cutaneous circulation is increased, and frictions with pressure upward, whereby the circulation is hastened. On the other hand, should the circulation be too rapid, inducing additional congestion of the lungs, and deepening the asphyxia, our remedy is the cold bath, whereby we constringe the cutaneous capillaries, retard the circulation, and diminish the pulmonary and cardiac congestion. We at the same time prevent the too rapid carbonization of the blood in the systemic circulation, thus partially arresting the generation of carbonic acid, which becomes a blood-poison when not eliminated by the respiratory process. Both the warm and the cold baths are also useful as stimulants to arouse the energies and excite the reflex actions of the nervous system.

If, however, we should not succeed by the process above given, we must have recourse to the cold bath. All animals bear a diminution of respiration in proportion to the diminished force and frequency of the circulation, and these latter phenomena are induced by cold, externally applied. Hibernating animals will scarcely drown at all, and warm-blooded animals drown much sooner in warm than in cold water. These facts, first announced by Dr. Haighton and Sir Anthony Carlisle, (Blundell, *op. cit.*, pp. 116, 117,) and afterwards confirmed by Milne-Edwards, Brown-Séquard and Marshall Hall, taken in connection with the experiments and conclusions of Legallois, given in the February number of this *Journal*, all have an important bearing upon this subject. They show that if we would preserve the viable asphyxiated neonatus, we must bear in mind the law of the correspondence between the circulation of the blood and the respiratory process, and must direct our efforts to the maintenance of respiration, whereby we decarbonize the blood, and control the circulation, by the warm and cold baths and frictions, into correspondence with the respiration which we execute.

